# The FT-IR and Raman Spectroscopies as Tools for Biofilm Characterization Created by Cariogenic Streptococci

**DOI:** 10.3390/ijms21113811

**Published:** 2020-05-27

**Authors:** Barbara Gieroba, Mikolaj Krysa, Kinga Wojtowicz, Adrian Wiater, Małgorzata Pleszczyńska, Michał Tomczyk, Anna Sroka-Bartnicka

**Affiliations:** 1 Department of Biopharmacy, Medical University of Lublin, Chodzki 4a, 20-093 Lublin, Poland; barbaragieroba@umlub.pl (B.G.); krysamikolaj@gmail.com (M.K.); kingaewawojtowicz@gmail.com (K.W.); 2 Department of Industrial and Environmental Microbiology, Institute of Biological Sciences, Maria Curie-Skłodowska University, Akademicka 19, 20-033 Lublin, Poland; adrianw2@poczta.umcs.lublin.pl (A.W.); m.pleszczynska@poczta.umcs.lublin.pl (M.P.); 3 Department of Pharmacognosy, Faculty of Pharmacy, Medical University of Białystok, ul. Mickiewicza 2a, 15-230 Białystok, Poland; michal.tomczyk@umb.edu.pl; 4 Department of Genetics and Microbiology, Maria Curie-Skłodowska University, Akademicka 19, 20-033 Lublin, Poland

**Keywords:** bacterial polysaccharides, FT-IR microspectroscopy, Raman spectroscopy, biofilms, dental caries, bacteria, mutans streptococci

## Abstract

Fourier transform infrared (FT-IR) and Raman spectroscopy and mapping were applied to the analysis of biofilms produced by bacteria of the genus Streptococcus. Bacterial biofilm, also called dental plaque, is the main cause of periodontal disease and tooth decay. It consists of a complex microbial community embedded in an extracellular matrix composed of highly hydrated extracellular polymeric substances and is a combination of salivary and bacterial proteins, lipids, polysaccharides, nucleic acids, and inorganic ions. This study confirms the value of Raman and FT-IR spectroscopies in biology, medicine, and pharmacy as effective tools for bacterial product characterization.

## 1. Introduction

Dental caries is an infectious disease associated with the accumulation of bacterial plaque on the tooth surface [1]. Dental plaque, the biofilm formed on the tooth surface, consists of a complex microbial community (less than 10% of biofilm dry weight) embedded in a bacterial and salivary-origin matrix of highly hydrated extracellular polymeric substances (EPS, more than 90% of biofilm dry weight). Dental plaque formation is a multistep process, which involves acquired pellicle formation, initial sucrose-independent and subsequent polysaccharide-mediated attachment of cells to the tooth surface, biofilm maturation, and dispersion of biofilm cells [2]. Mutans streptococci (MS, mainly *Streptococcus mutans* and *S. sobrinus*) have been isolated from human dental plaque and have been implicated as a primary causative agent of dental caries [3]. Adhesion, acidogenicity, and acid tolerance are the main virulence factors of the bacteria. MS secrete constitutive glucosyltransferases (Gtfs) that cooperatively synthesize polysaccharide components of EPS from ingested sucrose. *S. mutans* produce three glucosyltransferases. GtfD synthesizes water-soluble (1→6)-α-d-glucans (its activity is primer-dependent), GtfB and GtfC synthesize water-insoluble (1→3)-α-d-glucans, and the second produces a mixture of water-soluble and water-insoluble glucans, respectively [4,5,6]. It has been found that simultaneous synthesis of glucans by GtfB and GtfC is essential for formation of high-density biofilm with high adhesion, which promotes their binding to an apatite surface [7]. *S. sobrinus* strains extracellularly produce at least four kinds of Gtfs: (1→3)-α-d-glucan synthase (GtfI) and (1→6)-α-d-glucan synthase (GtfU), (1→6)-α-d-glucan synthase (GtfT), and an oligo-isomaltosaccharide synthase (GtfS) [8]. Glucosyltransferases and their polysaccharide products have been shown to be fundamental virulence factors in the pathogenesis of dental caries because they are responsible for close adhesion to the tooth surface in the presence of sucrose. Additional virulence factors of mutans streptococci are glucan-binding proteins (Gbps). *S. mutans* produces at least four Gbps: GbpA, GbpB, GbpC, and GbpD. The importance of these proteins is to maintain biofilm architecture by linking bacteria and extracellular molecules of glucan [9]. Another factor that is associated with the virulence of *S. mutans* is the cell surface protein antigen c (PAc). PAc participates in sucrose-independent bacterial adherence to the tooth surface via interaction with the salivary pellicle [7].

Fourier transform infrared (FT-IR) and Raman spectroscopies are powerful techniques for generating direct information about the molecular and chemical composition of biological samples [10,11,12]. Compared with conventional histological and/or microscopic methods, the microspectroscopic approach is considered advantageous because it is fast, non-invasive, staining- and labeling-free, and less susceptible to human subjective analysis. The combination of these complementary spectroscopic techniques can offer a more comprehensive approach to the analysis of intact samples and ensures more detailed chemical information [13]. The coupling of FT-IR or Raman vibrational spectrometers with a microscope can provide useful information on molecular differences and spatial distributions within and between various healthy and pathological cells and tissues at a microscopic level [13,14,15]. A recent Raman spectroscopic study on bacterial biofilms demonstrated that this technique enables the identification and analysis of nucleic acids, carbohydrates, proteins, and extracellular polymeric substances in biofilms created by a *Pseudomonas* sp. strain [16]. It has also been applied to characterization of the typology and matrix composition of biofilm produced by *Pseudoalteromonas haloplanktis* TAC125 in the context of environmental and cold adaptation [17]. In the field of dentistry, it can be used to assess the mineral properties of calcified tissue [18], examine the hydroxyapatite single crystallites [19], compare dental tissues, including enamel and dentin [20], and characterize hydroxylated phosphates [21]. It can also be applied to dental material research, [22,23] and finally utilized for tooth caries diagnosis [24,25].

The aim of the present study was to investigate the composition (the molecular structure and distribution of particular chemical components) of bacterial biofilms produced by various cariogenic strains of *Streptococcus spp.* with the use of FT-IR and Raman spectroscopic imaging. This vibrational spectroscopic approach proves to be useful in determining the structure of biological samples, which could potentially reduce the cost of experiments and shorten the time of analysis.

## 2. Results

### 2.1. FT-IR Spectroscopy

**Figure 1:**
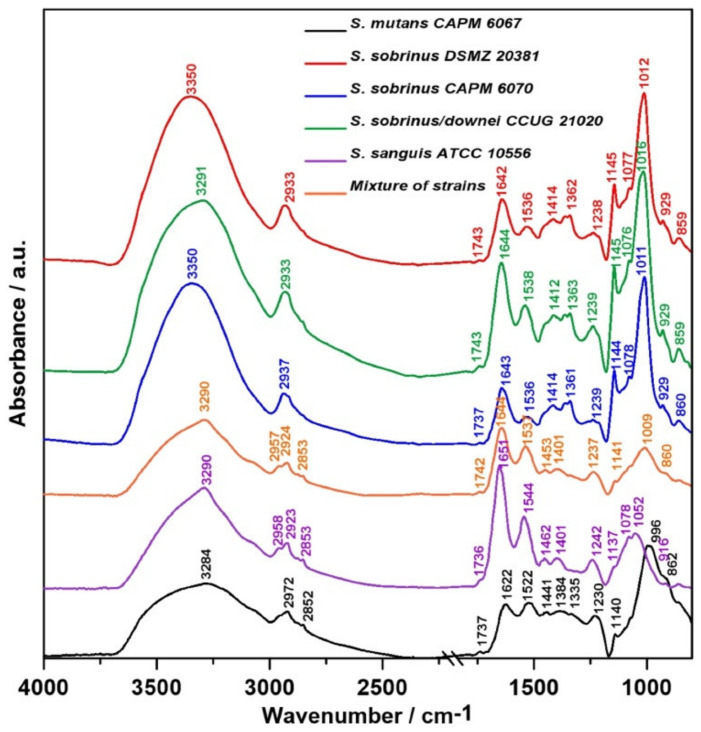
Representative relative intensity of Fourier transform infrared (FT-IR) spectra of bacterial biofilms.

**Figure 2:**
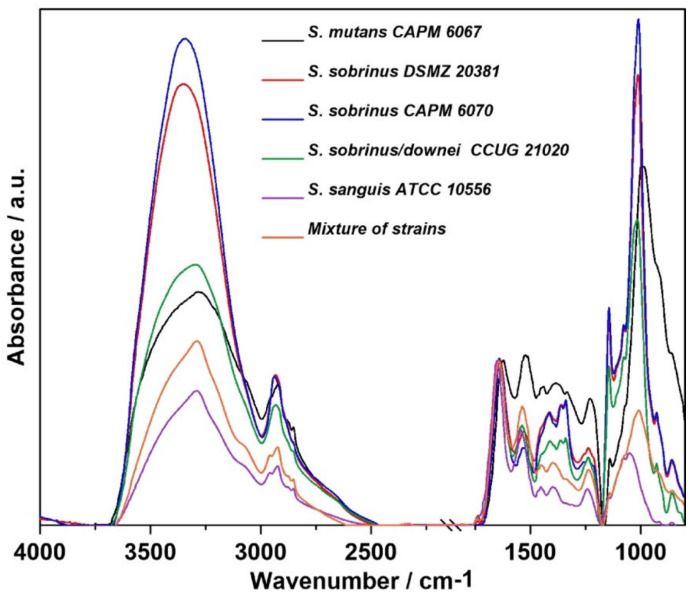
The FT-IR spectra of bacterial biofilms normalized to the Amide I band.

**Figure 3:**
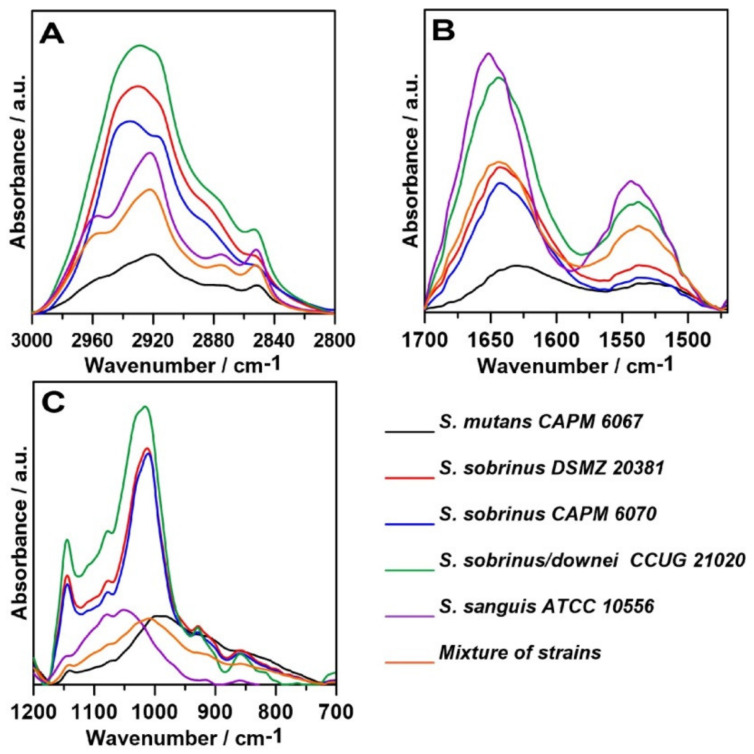
The relative intensity of FT-IR spectra of the fingerprints regions of: (**A**)—lipid region (3000–2800 cm^−1^), (**B**)—Amides I and II region (1700–1470 cm^−1^), and (**C**)—carbohydrate region (1200–700 cm^−1^).

The relative intensity and FT-IR spectra normalized to the Amide I band of the studied biofilm samples are presented in Figure 1 and Figure 2, respectively. A closer look at the regions of lipids, proteins, and carbohydrates shows the relative intensity of the FT-IR spectra, as shown in Figure 3.

The main changes between the samples have been shown in the protein, lipid, and sugar regions of averaged spectra, as shown in Figure 1, and normalized spectra, as presented in Figure 2. Table 1 summarizes the characteristic wavenumbers together with the proposed vibrational modes ascribed to functional groups in individual biofilm components [26,27,28,29,30,31,32,33,34,35,36,37]. The broad band at 3200–3350 cm^−1^ corresponds to Amide A [26] with strong absorbance intensity. Naumann et al. divided spectra acquired from bacterial samples into five so-called windows of vibration groups [27,38]. The spectral region of 3000–2800 cm^−1^ (Figure 3A) is assigned to symmetric and asymmetric vibrations of C—H in the —CH_2_ and —CH_3_ methylene groups, which are characteristic for lipids concentrated in bacterial cell walls and membranes [39]. The highest intensity of this band was detected for *S. sobrinus/downei* CCUG 21020 and the lowest for *S. mutans* CAPM 6067. The magnitude of these alterations may depend on the hydrocarbon chain length and chemical structure of the polar head group of the membrane lipids [40]. 

The Amide I and Amide II bands are susceptible to changes in the secondary structures of proteins [41]. Absorbance bands characteristic for Amide I and Amide II (Figure 3B, 1500–1700 cm^−1^) are relatively higher in *S. sanguis* ATCC 10556 and *S. sobrinus/downei* CCUG 21020 compared to other studied groups, and are the lowest in *S. mutans* CAPM 6067. Only small, inconsiderable shifts were observed in Amide I (1642–1651 cm^−1^) and Amide II bands (1536–1544 cm^−1^) in all spectra, except in *S. mutans* CAPM 6067 (1622 cm^−1^ and 1522 cm^−1^, respectively). It is worth to remember that in FT-IR measurements, water can distort the results, contributing to the Amide A and Amide I bands. Though bacterial biofilms have different densities, the overlapping of water may vary, reducing the analytical value of FT-IR spectroscopy in this case. Therefore, the results should be interpreted with caution [42]. 

The “sugar region” (Figure 3C), according to Naumann et al. [38] (1200–950 cm^−1^), is a spectral region that is important for the structural characterization of polysaccharides with the 1137–1144 cm^−1^ band, indicating the presence of oligosaccharides in all tested samples [34,36]. In *S. sobrinus* DSMZ 20381, *S. sobrinus* CAPM 6070, and *S. sobrinus/downei* CCUG 21020 samples, there is band at 1009–1016 cm^−1^ with a shoulder at 1076, 1077, and 1078 cm^−1^ assigned to β-glucan bonds [37,43]. The shoulder band at 1078 cm^−1^ also appeared in the *S. sanguis* ATCC 10556 sample. Moreover, the band characteristic for the common bacterial polysaccharide -(1→3),(1→6)-α-d-glucan (852–860 cm^−1^ range) is located in the “anomeric region” (950–700 cm^−1^) [37]. The latter region (1180–960 cm^−1^) contains weak bands that are sensitive to d-glucose [44]. In the tested samples, the highest intensity for glucose is in the range 999–1046 cm^−1^. The band at 929 cm^−1^ corresponds to (1→3)-α-d-glucan [43]. It is worth to remember that carbohydrates, phosphates/phospholipids, and nucleic acids have overlapping biological signals (the wavenumber region 1300–900 cm^−1^) in the mid-infrared (IR) [35]. 

**Figure 4:**
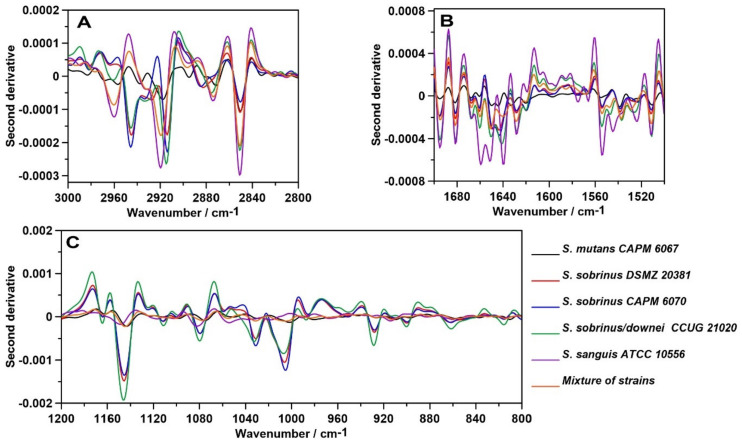
The second-order derivatives of the FT-IR spectra of biofilms; (**A**)—lipid region; (**B**)—protein region; (**C**)—carbohydrate region.

Next, the second-order derivatives were determined in three analyzed spectral regions (Figure 4). The aim of this operation was to study the molecular modifications of lipids, carbohydrates, and secondary structures of proteins in the tested biofilms.

The second-order derivative in the 3000–2800 cm^−1^ spectral range (Figure 4A) shows slight differences in the lipid profiles of the samples. The most pronounced variation is the ~2920 cm^−1^ shifts, which correspond to the stretching asymmetrical vibration of CH_2_ groups [45]. This suggests different lipid compositions of bacterial membranes in the analyzed strains.

The greatest similarity shows the course of the second derivative determined for Amide bands (Figure 4B). Only in the case of *S. sanguis* ATCC 10556, additional minima of the second derivative in 1648–1660 cm^−1^ assigned to α-helices and 1639 cm^−1^ corresponding to a parallel β-sheet secondary structure of proteins [46] were detected. This strain may produce adjective proteins contributing to biofilm adhesion and architecture or bacterial virulence.

The course of the second derivative function in terms of sugars (Figure 4C) indicates similarity in the carbohydrate composition of biofilms produced by the *S. sobrinus* DSMZ 20381, *S. sobrinus* CAPM 6070, and *S. sobrinus/downei* CCUG 21020 strains. Similarities in the polysaccharide region were also observed in *S. mutans* CAPM 6067, *S. sanguis* ATCC 10556, and mixes of strains. This indicates different carbohydrate profiles of the studied bacterial biofilms.

### 2.2. FT-IR Imaging 

**Figure 5:**
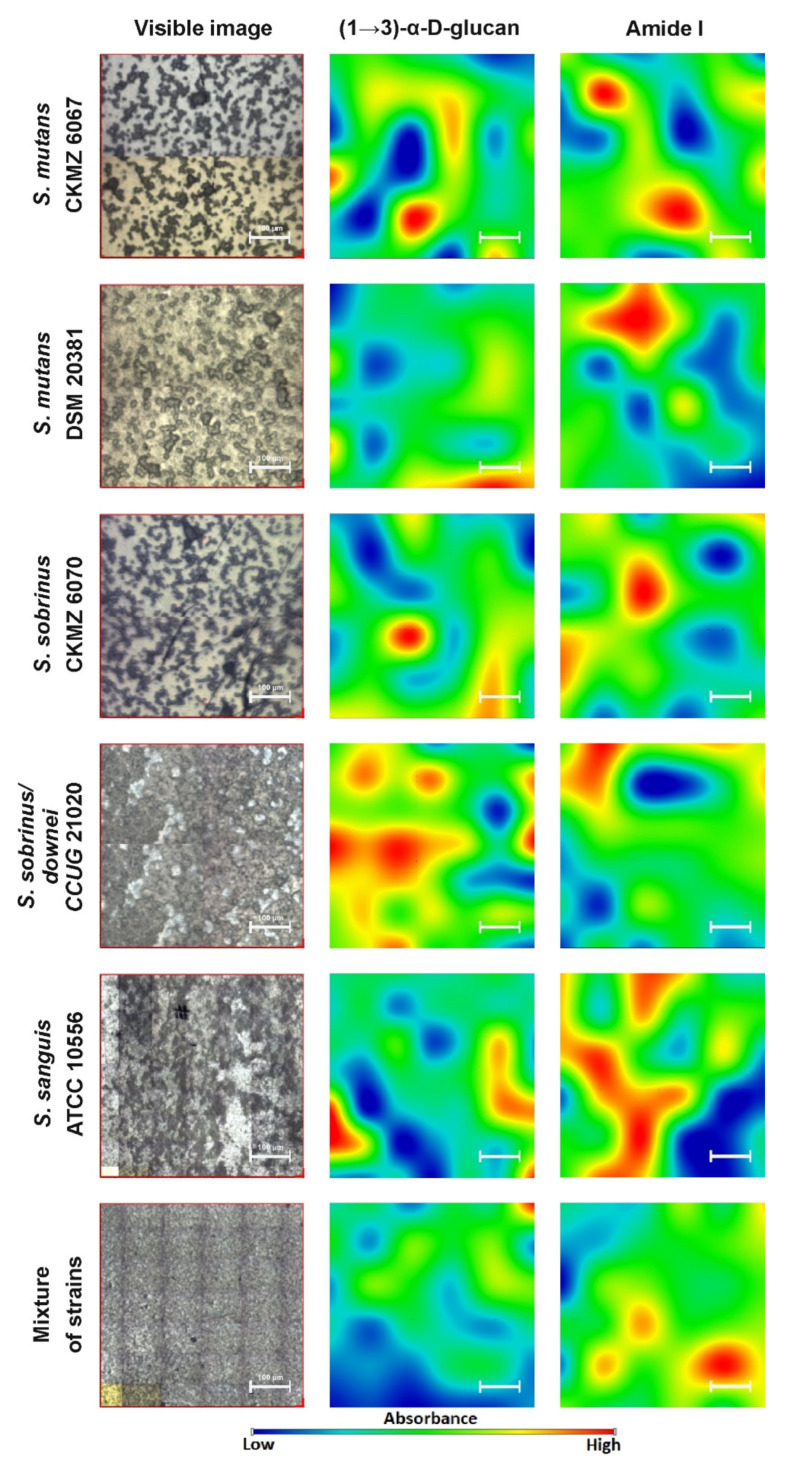
The FT-IR chemical maps of compound distributions in bacterial biofilms. The white bar corresponds to 100 µm.

Our next analysis was the spectroscopic mapping of the distribution of proteins and sugars in the biofilm samples. We did not examine the lipid region due to its smaller contribution in plaque adhesion, dispersion, and virulence potential. The results are presented in Figure 5.

The visualization (Figure 5) shows the spatial distribution and intensity of absorption of two important biofilm compounds ((1→3)-α-d-glucan and Amide I) in the x-y axis of the sample images. A magnified picture of each sample was taken prior to analysis and represented the differential structures of selected protein and carbohydrate bands. The presence of the bands, which are assigned to the (1→3)-α-d-glucan and Amide I (at 980–900 cm^−1^ and 1700–1600 cm^−1^, respectively), is visible in all samples, and confirms that they are characteristic biofilm components. The main visible difference between the chemical images is the lower absorbance intensity of the (1→3)-α-d-glucan bands in the *S. sobrinus* DSMZ 20381 and strain mixture samples. Regarding the Amide I band, the highest absorbance intensity is shown by the *S. sanguis* ATCC 10556 sample. The differences in intensity result from the various quantitative contents of the above-mentioned compounds in the studied bacterial biofilms. They are composed of various qualitative and quantitative sugar and protein constituents, which, in diversified ways, contribute to plaque formation and consequently influence the potential for dental caries progression. This may mean that each strain of bacteria creates a different type of biofilm, including in terms of density and the grade of adhesion.

### 2.3. Raman Spectroscopy

**Figure 6:**
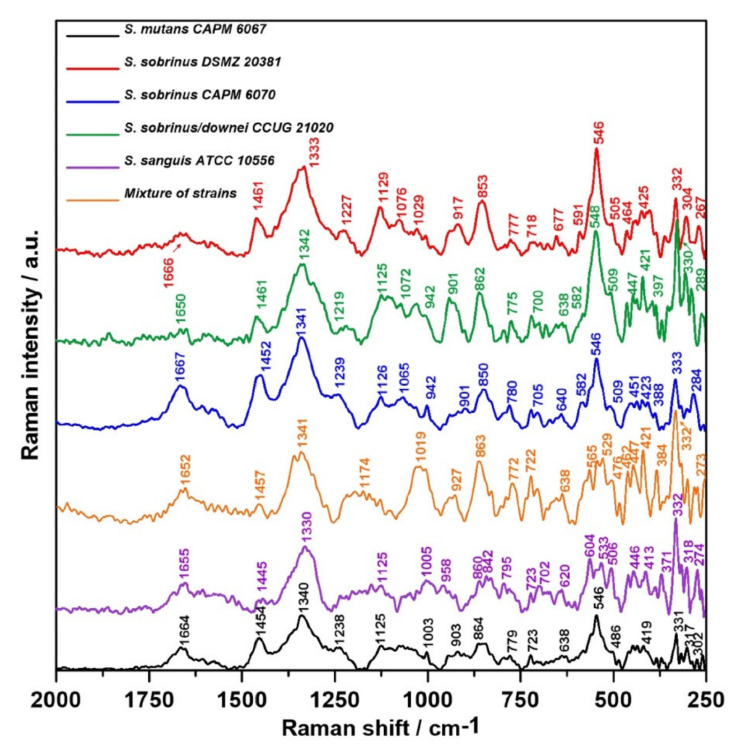
Representative relative intensity of the Raman spectra of bacterial biofilms.

**Figure 7:**
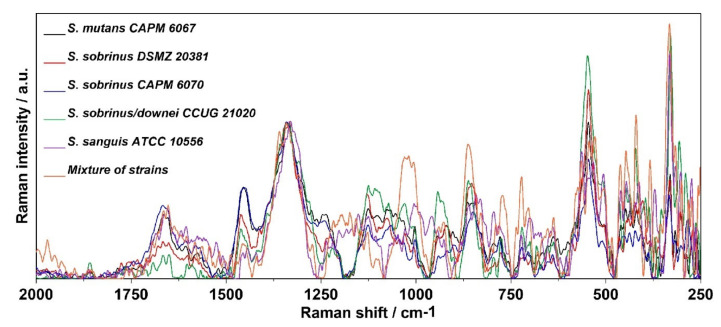
The Raman spectra normalized to the 1300–1400 cm^−1^ band (assigned to deformation vibration of the CH_2_ group in lipids and proteins) of bacterial biofilms.

**Figure 8:**
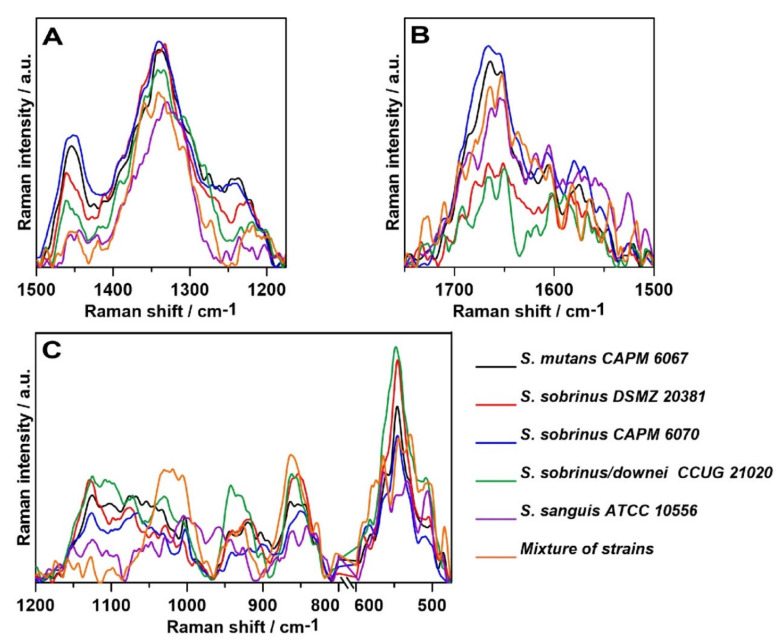
The relative intensity of the Raman spectra of the fingerprint region spectra of: (**A**)—lipid and Amide III region (1500–1175 cm^−1^), (**B**)—Amides I and II region (1750–1500 cm^−1^), and (**C**)—carbohydrate region (1200–800 and 610–475 cm^−1^).

The relative intensity and Raman spectra normalized to the 1300–1400 cm^−1^ band (mainly associated with vibrations of the CH_2_ group present in proteins, but also in lipids [47]) of the bacterial biofilm specimens are depicted in Figure 6 and Figure 7, respectively. A more accurate analysis of the regions of lipids, proteins, and carbohydrates is facilitated by the relative intensity of the Raman spectra presented in Figure 8.

Both in the FT-IR and in Raman spectra (Figure 6 and Figure 7), the main differences were related to the lipid, Amide, and carbohydrate regions. The full information of all of the vibrational modes present in the Raman spectra is collected in Table 2 [47,48,49,50,51,52,53,54,55,56,57,58,59,60,61]. The Raman spectra of lipids attributed to the presence of the hydrocarbon chain are mainly detected in the three following spectroscopic regions: 1500–1400 cm^−1^, 1300–1250 cm^−1^, and 1200–1050 cm^−1^ [48].

The highest intensity of the 1445–1461 cm^−1^ band assigned to saturated lipids [54] was observed for *S. sobrinus* CAPM 6070 and *S. mutans* CAPM 6067, and the lowest for *S. sanguis* ATCC 1056 (Figure 8A). Differences in the intensity of the previously mentioned band indicate alterations in lipid amounts and compositions of bacterial biofilms during maturation in terms of saturation of lipid fatty acids. 

In the spectra of the bacterial biofilms, Amide VI bands could be distinguished (Figure 6). Each of the above bands was observed clearly in all of the biofilm spectra; however, the intensities of the bands varied significantly among them, and the most significant peak was determined as Amide I. The lowest intensity of the Amide I and II bands showed *S. sobrinus* DSMZ 20381 and *S. sobrinus/downei* CCUG 21020 (Figure 8B), and that of the Amide III was detected in *S. sanguis* ATCC 10566 (Figure 8A). The highest intensity of the Amide I and III bands was noted in *S. mutans* CAPM 6067 and *S. sobrinus* CAPM 6070 (Figure 8A,B). This may suggest different protein compositions of the biofilms created by the studied strains, and thus other adhesive and caries-forming properties.

The shifting of the wavenumber position of the (1→6)-α-glycosidic bond band (852 cm^−1^) is associated with the methylesterification degree [59]. The most specific bands for glucans are those centered at ~757 and 520 cm^−1^. The ~380 cm^−1^ band can be ascribed to the β-d-glucosides (Figure 6). In Gram-positive cells, such as *Streptococcus* spp., the presence of the bands observed at 880–980, 1055, and 1075 cm^−1^ could arise from a combination of the vibrational modes of rhamnose, galactose, and glucose of bacterial cell walls [60]. Other authors report that the specific Raman peaks of glucose are at 1125 cm^−1^ [49,61]. The spectral range 790–950 cm^−1^ can be assigned to the side-group deformations of biofilm-characteristic carbohydrates [50]. The sugar profile in the area of 475–600 cm^−1^ for all samples has a similar course (Figure 8C); significant differences occur in the ~1020 and ~850 cm^−1^ bands in the mixed-strain specimen and in the ~950 cm^−1^ band in *S. sobrinus/downei* CCUG 21020, where the highest intensity was recorded. These can be interpreted as the differences in glucan and glucose contents.

Other spectral regions are responsible for the occurrence of these three main biofilm components as well. The Raman bands at ~1380 and ~1280 cm^−1^ are frequently considered as a polyanionic polysaccharide signature in the bacterial biofilm matrix [51,52]. Moreover, the occurrence of the phenylalanine ring breathing band could be utilized as a protein marker of a biofilm [47,51].

The polysaccharide, protein, and lipid Raman spectral ranges partially overlap with those of the nucleic acids, especially the DNA region. 

**Figure 9:**
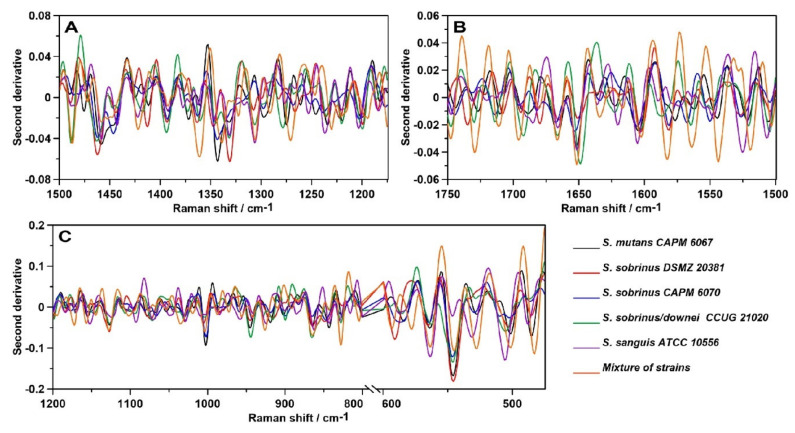
The second-order derivatives of the Raman spectra of biofilms; (**A**)—lipid region; (**B**)—protein region; (**C**)—carbohydrate region.

Subsequently, the second-order derivatives were determined in the three studied spectral regions (Figure 9) attributed to lipids (A), proteins—precisely, Amides I and II (B)—and polysaccharides (C). Hereby, more information can be drawn regarding the differences in the molecular structures of these components in analyzed bacterial biofilms.

The second-order derivative of the Raman spectra in the 1500–1175 cm^−1^ spectral range (Figure 9A) shows considerable differences in the lipid and Amide III profiles of the samples. The most noticeable variation is the ~1350 cm^−1^ shift, which corresponds to bending vibrations of –CH_2_ and –CH_3_ [45]. In addition, the ~1275 cm^−1^ shift can indicate alterations in the α-helical structure of polyprotein chains, and the ~1425 cm^−1^ shift can be assigned to the C–N stretching vibration [62]. The protein–lipid composition of the cell membrane may vary depending on the strain.

In addition, the course of the second derivative determined for the Amide bands significantly differs (Figure 9B). The most similarities can be observed in the mixture of strains and *S. sobrinus/downei* CCUG 2120. Moreover, in the case of *S. sanguis* ATCC 10556, additional shifts were detected: 1506, 1525, 1605, and 1620 cm^−1^, attributed to aggregates and antiparallel β-sheets in Amide II [63] and Amide I [46], respectively. Among all secondary structures of proteins, the β-sheet has the greatest diversity of functions. They influence the enzymes, antibodies, transport, or membrane protein functions, and may be crucial for virulence [62]. 

The course of the second derivative function with respect to sugars (Figure 9C), especially in the 1200–800 cm^−1^, region indicates similarity in the carbohydrate compositions of the analyzed biofilm samples. The biggest differences, particularly marked in the 600–475 cm^−1^ range, were detected in the mixture of strains and in the *S. sobrinus/downei* CCUG 21020 sample. The 527–531 cm^−1^ and 502–506 cm^−1^ shifts in these strains are correlated with glucans and d-xylose [64] structures, respectively. These data are in accordance with the results presented in Figure 8C, indicating the variant compositions of glucans and other sugars.

### 2.4. Raman Imaging 

**Figure 10:**
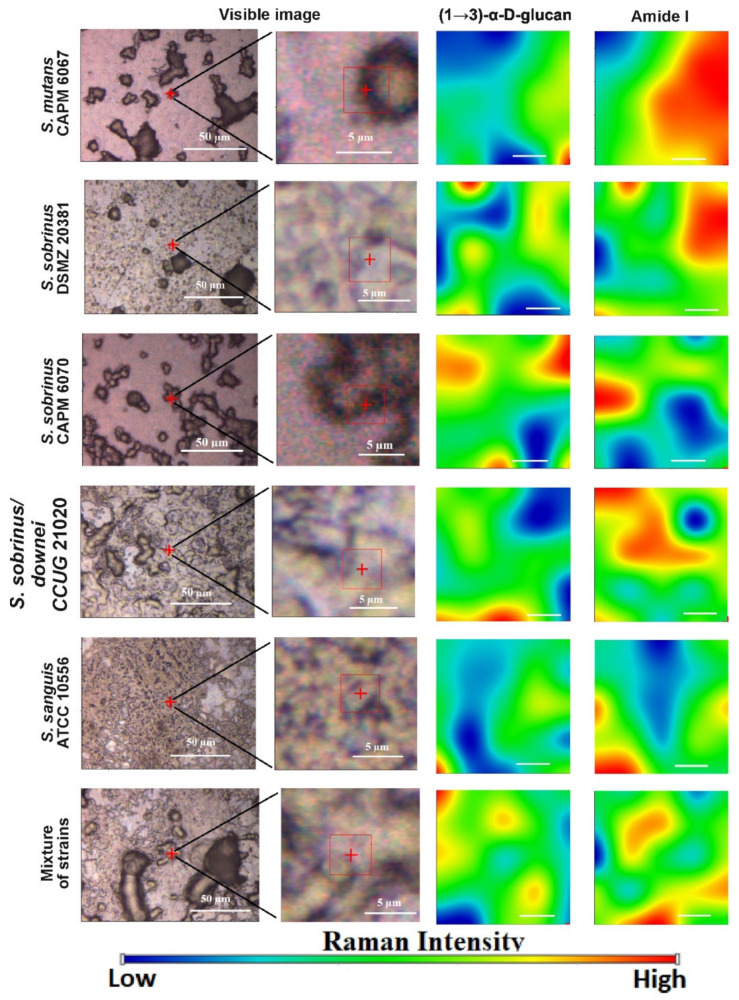
The Raman chemical maps of compound distributions in bacterial biofilms. The red cross and frame in the optical images indicate the mapping area. The white bar in the chemical maps corresponds to 1 µm.

Similarly to the FT-IR imaging, we performed Raman imaging for the same components, sugars and proteins. The results are presented in Figure 10.

Identically to FT-IR microspectroscopy, all studied components were present in the Raman imaging visualization. Chemical analysis (Figure 10) confirmed different spatial distributions of (1→3)-α-d-glucan and Amide I in the tested bacterial biofilms. The lowest content of (1→3)-α-d-glucan was detected in the *S. sanguis* DSMZ 20381 and *S. sanguis* ATCC 10556 samples. In all biofilm samples formed by the strains selected for study, the content of Amide I was quite high.

Collectively, FT-IR and Raman imaging study proved that less (1→3)-α-d-glucan in bacterial biofilms was formed by the *S. mutans* CAPM 6067, *S. sanguis* ATCC 10556, and *S. sobrinus/downei* CCUG 21020 strains. Furthermore, the amount and distribution of Amide I differs slightly among the probes. 

## 3. Discussion

Generally, biofilms are described as “complex communities of bacteria residing within an exopolysaccharide matrix that adheres to a surface” [65]. Biofilm production by bacterial strains is a significant medical and clinical problem because it may be the cause of chronic disease or infections from hospitals, and may be related to infections from implantable medical devices (i.e., dialysis catheters, artificial heart valves, heart Pacemakers, drainage tubes, orthopedic prostheses) [66]. In dentistry, biofilms contribute mainly to dental plaque formation, which, in turn, leads to tooth caries and chronic gingivitis. Biofilms also contribute to infection in the para-nasal sinuses and adhere to dental prostheses and implants, constituting a particular risk for patients with impaired immunity [67]. The phenomenon of quorum sensing—the way bacterial cells communicate with each other, determining surface adhesion, EPS, and virulence factor production—is involved in the formation of bacterial biofilms [68]. EPSs are mainly polysaccharides and, in the matrix of the dental plaque, mostly occur as glucose homopolymers, such as (1→3)-α-, (1→4)-α-, (1→6)-α-d-glucans, while (1→3),(1→6)-α-d-glucan remains crucial for dental caries ethology [2]. The spectral analysis of glucans in biofilms is difficult due to the fact that the presence of these components is attributed to several wavenumbers in both FT-IR and Raman spectra. Furthermore, carbohydrate bands overlap with those of other compounds, such as DNA/RNA, phosphorylated lipids, and proteins [35]. The specificity of glucans can be confirmed by other methods, e.g., polysaccharide-specific monoclonal anti-bodies [69]. FT-IR spectra revealed that a greater amount of (1→3),(1→6)-α-d-glucan is contained in biofilms formed by *S. sobrinus* DSMZ 20381 and *S. sobrinus* CAPM 6070. They also exhibit a similar profile to that of other polysaccharides, and may have a higher caries-forming potential than other tested streptococci strains. Raman spectra confirmed the differences in glucans and xylose content and the lower glucose quantity in *S. sobrinus*/*downei* CCUG 21020 and the mixture of strains. The FT-IR and Raman chemical images presented in Figure 5 and Figure 10 show the specific component distribution within the measured area. The distributions of the Amide I and (1→3)-α-d-glucan band vary between the different samples, indicating different cariogenic potential.

So far, it was confirmed that mixed bacterial species’ biofilms produce considerably more biomass compared with biofilms of one bacterial species, with no need to provide additional external nutrients [70,71]. Moreover, it was stated that these complex bacterial communities have unique properties, e.g., greater resistance to antimicrobial agents and chemical stress/substances, as well as superior expansiveness [70,72]. In our study, the biofilm formed by the mixture of strains has a very similar composition to those of *S. mutans* CAPM 6067, *S. sanguis* ATCC 10556, and *S. sanguis/downei* CCUG 21020 biofilms, while it was characterized by a different polysaccharide content, what can contribute to its different features. 

Lipids represent only circa 1.8% of the biofilm matrix [73], but can lead to binding to metals (e.g., in dental prostheses), enhancing virulence, and increasing microbial adherence together with lipopolysaccharides [74]. It was proven that strongly adherent microbial cells increased the production of saturated membrane lipids [75]. They may also play a role as biosurfactants, like viscosin, surfactant, and emulsan, which enable the bioavailability of dispersed hydrophobic substances [76]. Due to the low content of lipids in bacterial biofilms, we did not study their spatial distribution by FT-IR and Raman microspectroscopies, but we observed that lipid composition varied in the studied strains, especially in the ~2920 cm^−1^ FT-IR band, influencing the cell membrane saturation. In combination with the sugar composition, it may indicate different adhesion potential. *S. mutans* CAPM 6067, *S. sanguis* ATCC 10556, and the mixture of strains had the most similar lipid profile, but also sugar profile, and thus probably have congenial adhesive properties.

Protein in the extracellular matrix mainly has two different functions, depending on the location—as enzymes and virulence factors [77]. Enzymatic proteins are involved in the degradation of water-soluble (proteins, nucleic acids, and polysaccharides) and insoluble (lipids) organic components prevalent in biofilms. They promote cell dispersion and, therefore, colonization of new areas [73]. Virulence agents participate in infection processes, including within the oral cavity [78]. Moreover, it was described that *S. mutans* CAPM 6067 produces glucan-binding proteins, like lectins, leading to formation and stabilization of the matrix [9]. In our research, in terms of protein content, biofilm produced by *S. sanguis* ATCC 10556 significantly stands out. In the FT-IR spectrum, a higher absorbance intensity was recorded, a different secondary structure of proteins was detected, and a much higher content of Amide I distribution in spectral mapping was found. In the Raman studies, alteration in the β-sheet secondary structure was also revealed. This may testify for the higher colonization potential of this strain.

The quantification of various cellular structures like lipids, proteins, and sugars evidences not only the modifications in the metabolism of bacterial cells and bacterial products, but can also serve as a potential marker for cariogenic processes [79]. FT-IR and Raman spectroscopies are very efficient tools applied for the detection, characterization, and analysis of the above-mentioned molecules. These techniques remain an attractive approach because they are cheap and do not require additional reagents, high-grade solvents, or expensive internal standards and equipment. Moreover, they are widely accessible in standard basic laboratories, and these techniques are potent and adequate for routine studies [39].

Although FT-IR and Raman microspectroscopies have been commonly used as potential techniques for analysis of metabolic profiles of cells and their products in biomedical science [80], they have some limitations. Due to their high complexity connected with overlapping or broadened signals from different simultaneously absorbing cellular components, sometimes, it becomes a problem to ascribe variations in absorbance at a particular wavenumber to a specific molecule [35,81]. To overcome this difficulty, advanced mathematical methodologies for spectral data analyses [82,83], such as second-order derivative determination, Gaussian and/or Lorentzian curve fitting, and Voight deconvolution, can be applied [84]. In our case, the second-order derivative function proved to be sufficient because we compared the composition of the biofilms produced by various streptococci strains, not the changes in biofilm created by one bacterial strain during different conditions (such as drug treatment or alternating ion composition, pH, or temperature).

Even though FT-IR and Raman spectroscopies are complementary techniques that measure the vibrational energies of molecules, both methods are based on different selection rules—an absorption process and an inelastic scattering effect of electromagnetic radiation, respectively. Therefore, the combination of these complementary spectroscopies, as we did in this research, can offer a more comprehensive approach for analyzing intact samples, and can ensure more detailed chemical information [13]. The differences in the results obtained using both techniques are due to the different sensitivities to detection of particular chemical groups and types of vibrations; therefore, some overlapping or low-intensity bands can be distinguished only by one of these methods. For instance, by means of Raman spectroscopy, the most intensive bands are recorded from the symmetric, non-polar groups, e.g., C–C, C=C, C–S, and S–S, but, generally, vibrational spectroscopies are receptive to the anomeric configuration of glycosidic bonds. Additionally, the Raman scatter from water is relatively weak. [43]. In order to avoid water contribution problems, other techniques should be applied in the study of the biochemical composition of bacterial biofilms, which include, among others, the combination of IR and Raman with confocal scanning light microscopy (CSLM), small-angle x-ray scattering (SAXS), surface plasmon resonance imaging (SPRi), electrochemical surface plasmon resonance (EC-SPR), and microscopic approaches: Scanning electron microscopy (SEM) and atomic force microscopy [85].

In summary, our data demonstrate that FT-IR and Raman vibrational spectroscopies coupled with a microscopic approach can be utilized in combination with other biochemical techniques as additional determination and confirmation of the cariogenic potential of bacteria of the genus Streptococcus. The general technique we employed for biochemical analysis is applicable for investigating the bacterial cells’ inherence and proliferation, as well as their extracellular polymeric substance (EPS) production. This opens the possibility of applying non-invasive spectral optical techniques to monitor bacterial adhesion and biofilm production directly on tooth enamel, providing a valuable tool for measuring dental pathologies, such as caries, in vivo.

## 4. Materials and Methods 

### 4.1. Microorganisms

The streptococcal strains used for this study were: *Streptococcus mutans* CAPM 6067 and *S. sobrinus* CAPM 6070 (The Collection of Animal Pathogenic Microorganisms, Brno, Czech Republic), *S. sobrinus/downei* CCUG 21020 (The Culture Collection, University of Göteborg, Gothenburg, Sweden), *S. sanguis* ATCC 10556 (American Type Culture Collection, Manassas, VA, USA), and *S. sobrinus* DSMZ 20381 (DSMZ, German Collection of Microorganisms and Cell Cultures, Braunschweig, Germany).

### 4.2. Streptococcal Biofilm Formation

Six 100 mL flasks, containing 75 mL brain heart infusion broth (BHI) (BTL, Łódź, Poland) with 2% (*w*/*v*) sucrose, were autoclaved (30 min, 121 °C), and then 10^5^ CFU/mL test bacteria were inoculated into each flask. One flask was inoculated with a mixed culture of all cariogenic streptococci. Subsequently, a sterile aluminum-coated (thickness ~100 nm) Clear Borosilicate Float Glass Microscope Slide (DRLI, Deposition Research Lab Inc., St. Charles, MO, USA) was immersed in each flask, and batch cultures were incubated at 37 °C for 24 h under stationary conditions. After incubation, media and planktonic cells were removed, and the remaining biofilm adhering to the glass surface was rinsed with phosphate buffered saline (PBS). During the measurements, the samples were semi-dry biofilms.

### 4.3. FT-IR Microspectroscopy

FT-IR spectra were collected in a transflection mode with the use of a Nicolet 6700 FT-IR spectrometer (Thermo Scientific, Waltham, MA, USA) over the range 4000–600 cm^−1^. For each sample, five spectra under the same conditions were examined and averaged. Each spectrum represented 120 scans taken at a resolution of 4 cm^−1^ with an optimal signal-to-noise ratio. For a given material, a final spectrum was obtained using OMNIC 8.2.0.387 software (Thermo Fisher Scientific, Madison, WI, USA). Baseline corrections and further processing of spectra were performed using Grams Software and GRAMS/AI software (ThermoGalactic Industries, Keene, NH, USA). In order to trace the qualitative changes in the particular cellular components, such as lipids, proteins, and carbohydrates, the spectral mapping was performed in the appropriate spectral range (3000–2800 cm^−1^ for lipids, 1700–1470 cm^−1^ for proteins, 1200–700 cm^−1^ for sugars) after baseline and offset correction. To determine changes in these structures, second-order derivative spectra were calculated using the Savitzky–Golay algorithm with nine points.

For area mapping, the X and Y step size was 100 µm (4 × 9 points). The size of the imaging area of the sample was 400 × 900 μm, and the IR objective was ×15. Image assembly was performed using OMNIC 8.2.0.387 and CytoSpec (version 2.00.01, Berlin, Germany) software.

### 4.4. Raman Microspectroscopy

Raman spectra were recorded with the use of a DXR Raman Microscope (Thermo Scientific, Waltham, MA, USA). The excitation laser wavelength was 780 nm and the output power was set at 13 mW. The spectra were recorded in the 2000–250 cm^−1^ spectral range with 4 cm^−1^ of Raman shift resolution. A 25 μm pinhole aperture and exposure time of 6 s with 10 exposures per point with ×10 objective were used. The microscope was equipped with a CCD Camera (Sentech, Ebina, Kanagawa, Japan) and 0.8 mega-pixel CCD sensor. Mapping consisted of 875 single-measure points with a step size of 25 µm. The autofocus at each map point was used in the case of height-diverted samples. All data processing and image assembly was performed using OMNIC 8.2.0.387 (Thermo Fisher Scientific, Madison, WI, USA) and CytoSpec (version 2.00.01, Berlin, Germany) software. The five spectra from each sample were collected, baseline-corrected, and then averaged before analysis. In the study, the qualitative changes in the same cellular components as in FT-IR spectroscopy for the appropriate Raman fingerprint spectral ranges were chosen: 1500–1175 cm^−1^ for lipids, 1750–1500 cm^−1^ for proteins, and 1200–800 and 610–475 cm^−1^ for sugars). The spectra were baseline and offset corrected in this bandwidth. To determine changes in these structures, second-order derivative spectra were calculated using the Savitzky–Golay algorithm with thirteen points.

## 5. Conclusions

FT-IR and Raman spectra of bacterial biofilms provide information on the chemical profile of the sample. Testing does not require prior preparation; moreover, biological material can be tested both dry and wet. Spectroscopic spectra showed that bacterial biofilms consist mainly of a mixture of proteins and polysaccharides. Glucans, as detected during the research, plays an important role in the process of adsorption of pathogens to tooth enamel. This results in an increase in the mass of the bacterial biofilm and better adhesion of the colony to the tooth surface. This, in turn, intensifies carious processes and causes a violation of the enamel structure.

## Figures and Tables

**Table 1:** The most significant bands obtained in the type of FT-IR vibration with particular assigned components. Spectra of biofilm samples and the types of vibrations with particular assigned components.

Wavenumber (cm^−1^)	Assignment and the Type of Vibration *
3200–3350	ν (N–H), ν (O-H), Amide A, water
2950–2960	ν_as_ (CH_3_), lipids
2920–2940	ν_as_ (CH_2_), lipids
2850–2860	ν_s_ (CH_2_), lipids
1730–1740	ν (C=O), phospholipids
1700–1600	80% ν (C=O), 20% ν (C-N), τ (HOH), Amide I, water
1600–1500	60% τ (N–H), 30% ν (C–N), 10% ν (C–C), Amide II
1441–1462	pyrrolidine ring vibration of proline and hydroxyproline
1450–1400	δ_as_ (CH_3_), δ_as_ (CH_2_), proteins, lipids
1400–1350	δ_s_ (CH_3_), δ_s_ (CH_2_), ν_s_ (C=O), proteins, lipids
1350–1200	τ (N–H), ν (C–N), τ (C=O), ν (C–C), ν (CH_3_), Amide III,
1242–1230	ν_as_ (PO_2_^–^), DNA, RNA, phospholipids, phosphorylated proteins
1144–1137	Oligosaccharydes
~1086	ν_s_ (PO_2_^–^), DNA, RNA, phospholipids, phosphorylated proteins
1080–1070	ν (C–C), β-glucan bonds
1046–999	Skeletal vibration connected to anomeric structure of d-glucose
1009–1016	ν (C–C), RNA, ribose
~972	ν (C–C), ν (C–O), DNA, deoxirobose
900–700	anomeric ring vibrations for tryptophan, tyrosine, and phenyloalanine
929	(1→3)-α-d-glucan
860–852	(1→3),(1→6)-α-d-glucan

* Types of vibrations: stretching (ν), deformational (δ), bending (τ), symmetrical (s), and asymmetrical (as) modes.

**Table 2:** The most important bands obtained in the Raman spectra of biofilm samples and the types of vibrations with particular assigned components.

Raman Shift (cm^−1^)	Assignment and the Type of Vibration *
1700–1600	ν (C=O), Amide I
1667–1650	ν (C=C), lipids, proteins
1600–1500	ν (C–N), δ (N–H), Amide II
1576	adenine, guanine (DNA bases)
1523	cytosine (DNA bases)
1500–1400	in-plane τ and out-of-plane τ (CH_2_), lipids
1461–1445	ν_s_ (CH_2_), saturated lipids
~1380	δ (COH), (HCO), (HCC), ν_s_ (COO–), (C–O), polyanionic polysaccharide
1340–1330	polynucleotide chains, DNA purine bases
1330–1125	trans ν (C–C), lipids
1300–1250	in-plane τ and out-of-plane τ (CH_3_), lipids
~1280	δ (COH), (HCO), (HCC), ν_s_ (COO–), (C–O), polyanionic polysaccharide
1300–1230	ν (C–N), δ (N–H), Amide III
~1260 (shoulder band)	δ (CH), lipids, proteins
1200–1050	ν (C–C), lipids
1075, 1055, 980–880	combination of rhamnose, galactose, and glucose
~1127	ν (C–N), prolinę
1125	Glucose
~1120	ν_s_ (COC), glycosidic bonds
~1094	ν_as_ (COC), (1→4)-β-linked glycosidic bonds
~1068	trans ν (C–C), lipids
1000	phenyloalanine ring breathing
950–790	side group δ (COH), (C–CH), (O–CH), carbohydrates
~948	(1→3)-α-d-glucan
800–640	out-of-plane τ (N–H), Amide V
852	(1→6)-α-d-glucan
~783	ring breathing of cytosine, thymine, uracil; ν_s_ (O–P–O), phosphodiester bonds in DNA
770–625	τ (O=C-N), Amide IV
~757, ~520	Glucans
600–540	out-of-plane τ (C=O), Amide VI
~380	β-d-glucoside

* Types of vibrations: stretching (ν), deformational (δ), bending (τ), symmetrical (s), and asymmetrical (as) modes.

## Abbreviations

FT-IR	Fourier transform infrared spectroscopy
Gbps	Glucan-binding proteins
Gtfs	Glucosyltransferases
PAc	Cell surface protein antigen c
MS	Mutans streptococci

## References

[1] Marsh P.D. (2006). Dental diseases—Are these examples of ecological catastrophes?. Int. J. Dent. Hyg..

[2] Pleszczyńska M., Wiater A., Janczarek M., Szczodrak J. (2015). (1→3)-α-d-Glucan hydrolases in dental biofilm prevention and control: A review. Int. J. Biol. Macromol..

[3] Loesche W.J. (1986). Role of Streptococcus mutans in human dental decay. Microbiol. Rev..

[4] Aoki H., Shiroza T., Hayakawa M., Sato S., Kuramitsu H.K. (1986). Cloning of a streptococcus mutans glucosyltransferase gene coding for insoluble glucan synthesis. Infect. Immun..

[5] Hanada N., Kuramitsu H.K. (1989). Isolation and characterization of the Streptococcus mutans gtfD gene, coding for primer-dependent soluble glucan synthesis. Infect. Immun..

[6] Hanada N., Kuramitsu H.K. (2005). Isolation and characterization of the streptococcus mutans gtfc gene, coding for synthesis of both soluble and insoluble glucans. Infect. Immun..

[7] Matsumoto-nakano M. (2018). Role of Streptococcus mutans surface proteins for biofilm formation. Jpn. Dent. Sci. Rev..

[8] Nanbu A., Hayakawa M., Takada K., Shinozaki N. (2000). Production, characterization, and application of monoclonal antibodies which distinguish four glucosyltransferases from Streptococcus sobrinus. FEMS Immunol. Med. Microbiol..

[9] Lynch D.J., Fountain T.L., Mazurkiewicz J.E., Banas J.A. (2007). Glucan-binding proteins are essential for shaping Streptococcus mutans biofilm architecture. FEMS Microbiol. Lett..

[10] Carter E.A., Tam K.K., Armstrong R.S., Lay P.A. (2009). Vibrational spectroscopic mapping and imaging of tissues and cells. Biophys. Rev..

[11] Song C.L., Kazarian S.G. (2019). Three-dimensional depth profiling of prostate tissue by micro ATR-FTIR spectroscopic imaging with variable angles of incidence. Analyst.

[12] Song C.L., Vardaki M.Z., Goldin R.D., Kazarian S.G. (2019). Fourier transform infrared spectroscopic imaging of colon tissues: Evaluating the significance of amide I and C–H stretching bands in diagnostic applications with machine learning. Anal. Bioanal. Chem..

[13] Lin S., Li M., Cheng W. (2007). FT-IR and Raman vibrational microspectroscopies used for spectral biodiagnosis of human tissues. Spectroscopy.

[14] Kazarian S.G., Chan K.L.A. (2006). Applications of ATR-FTIR spectroscopic imaging to biomedical samples. Biochim. Biophys. Acta.

[15] Prince R.C., Potma E.O. (2019). Going visible: High-resolution coherent Raman imaging of cells and tissues. Light Sci. Appl..

[16] Henry V.A., Jessop J.L.P., Peeples T.L. (2017). Differentiating Pseudomonas sp. strain ADP cells in suspensions and biofilms using Raman spectroscopy and scanning electron microscopy. Anal. Bioanal. Chem..

[17] Ricciardelli A., Casillo A., Vergara A., Balasco N., Corsaro M.M., Tutino M.L., Parrilli E. (2019). Environmental conditions shape the biofilm of the Antarctic bacterium Pseudoalteromonas haloplanktis TAC125. Microbiol. Res..

[18] Imbert L., Gourion-Arsiquaud S., Villarreal-Ramirez E., Spevak L., Taleb H., van der Meulen M.C., Boskey A.L. (2018). Dynamic structure and composition of bone investigated by nanoscale infrared spectroscopy. PLoS ONE.

[19] Fu B., Sun X., Qian W., Shen Y., Chen R., Hannig M. (2005). Evidence of chemical bonding to hydroxyapatite by phosphoric acid esters. Biomaterials.

[20] Tsuda H., Ruben J., Arends J. (1996). Raman spectra of human dentin mineral. Eur. J. Oral. Sci..

[21] Bista R.K., Bruch R.F. (2009). Near-infrared spectroscopic studies of self-forming lipids and nanovesicles. Nanoscale Imaging, Sensing, and Actuation for Biomedical Applications VI.

[22] Liu Y., Yao X., Liu Y.W., Wang Y. (2014). A Fourier transform infrared spectroscopy analysis of carious dentin from transparent zone to normal zone. Caries Res..

[23] Hȩdzelek W., Wachowiak R., Marcinkowska A., Domka L. (2008). Infrared spectroscopic identification of chosen dental materials and natural teeth. Acta Phys. Pol. A.

[24] El-Sharkawyi Y.H. (2019). Detection and characterization of human teeth caries using 2D correlation raman spectroscopy. J. Biomed. Phys. Eng..

[25] Buchwald T., Buchwald Z. (2019). Assessment of the Raman spectroscopy effectiveness in determining the early changes in human enamel caused by artificial caries. Analyst.

[26] Baiz C.R., Reppert M., Tokmakoff A. (2013). Amide I two-dimensional infrared spectroscopy: Methods for visualizing the vibrational structure of large proteins. J. Phys. Chem. A.

[27] Zarnowiec P. (2015). Fourier Transform Infrared Spectroscopy (FTIR) as a tool for the identification and differentiation of pathogenic bacteria. Curr. Med. Chem..

[28] Lin H., Deng K., Zhang J., Wang L., Zhang Z., Luo Y., Huang P. (2019). Biochemical detection of fatal hypothermia and hyperthermia in affected rat hypothalamus tissues by Fourier transform infrared spectroscopy. Biosci. Rep..

[29] Hernadez B., Pfluger F., Adenier A., Kurglik S.G., Ghomi M. (2009). Vibrational analysis of amino acids and short peptides in hydrated media. IV. Amino acids with hydrophobic side chains: l-Alanine, l-Valine, and l-Isoleucine. J. Phys. Chem..

[30] Gao Y., Huo X., Dong L.I.U., Sun X., Sai H.E., Wei G., Wu J. (2015). Fourier transform infrared microspectroscopy monitoring of 5-fluorouracil-induced apoptosis in SW620 colon cancer cells. Mol. Med. Rep..

[31] Choo-Smith L.P., Maquelin K., Van Vreeswijk T., Bruining H.A., Puppels G.J., Thi N.N., Orsini F. (2001). Investigating Microbial (Micro) colony heterogeneity by vibrational spectroscopy. Appl. Environ. Microbiol..

[32] Gieroba B., Arczewska M., Sławińska-Brych A., Rzeski W., Stepulak A., Gagoś M. (2020). Prostate and breast cancer cells death induced by xanthohumol investigated with Fourier transform infrared spectroscopy. Spectrochim. Acta Part A Mol. Biomol. Spectrosc..

[33] Júnior Z.S.S., Botta S.B., Ana P.A., França C.M., Fernandes K.P.S., Mesquita-Ferrari R.A., Bussadori S.K. (2015). Effect of papain-based gel on type I collagen—Spectroscopy applied for microstructural analysis. Sci. Rep..

[34] Synytsya A., Novák M. (2013). Structural diversity of fungal glucans. Carbohydr. Polym..

[35] Sahu R.K., Salman A., Mordechai S. (2017). Tracing overlapping biological signals in mid-infrared using colonic tissues as a model system. World J. Gastroenterol..

[36] Yoshida S., Miyazaki M., Sakai K., Takeshita M., Yuasa S., Sato A., Okuyama H. (1997). Fourier transform infrared spectroscopic analysis of rat brain microsomal membranes modified by dietary fatty acids: Possible correlation with altered learning behavior. Biospectroscopy.

[37] Baeva E., Bleha R., Lavrova E., Sushytskyi L., Čopíková J., Jablonsky I., Synytsya A. (2019). Polysaccharides from basidiocarps of cultivating mushroom pleurotus ostreatus: Isolation and structural characterization. Molecules.

[38] Nauman D., Helm D., Labischinski H. (1991). Microbiological characterizations by FT-IR spectroscopy. Nature.

[39] Derenne A., Vandersleyen O., Goormaghtigh E. (2014). Lipid quantification method using FTIR spectroscopy applied on cancer cell extracts. Biochim. Biophys. Acta Mol. Cell Biol. Lipids.

[40] Shapaval V., Brandenburg J., Blomqvist J., Tafintseva V., Passoth V. (2019). Biotechnology for Biofuels Biochemical profiling, prediction of total lipid content and fatty acid profile in oleaginous yeasts by FTIR spectroscopy. Biotechnol. Biofuels.

[41] Barth A. (2007). Infrared spectroscopy of proteins. Biochim. Biophys. Acta Bioenerg..

[42] Sabbatini S., Conti C., Orilisi G., Giorgini E. (2017). Infrared spectroscopy as a new tool for studying single living cells: Is there a niche?. Biomed. Spectrosc. Imaging.

[43] Synytsya A., Novak M. (2014). Structural analysis of glucans. Ann. Transl. Med..

[44] Tanaka S., Kojić D., Tsenkova R., Yasui M. (2018). Quantification of anomeric structural changes of glucose solutions using near-infrared spectra. Carbohydr. Res..

[45] Shakeel F., Baboota S., Ahuja A., Ali J., Shafiq S. (2008). Skin permeation mechanism and bioavailability enhancement of celecoxib from transdermally applied nanoemulsion. J. Nanobiotechnol..

[46] Kong J., Yu S. (2007). Fourier transform infrared spectroscopic analysis of protein secondary structures protein FTIR data analysis and band assign. Acta Biochim. Biophys. Sin..

[47] Huang H., Shi H., Feng S., Chen W., Yu Y., Lin D., Chen R. (2013). Confocal Raman spectroscopic analysis of the cytotoxic response to cisplatin in nasopharyngeal carcinoma cells. Anal. Methods.

[48] Czamara K., Majzner K., Pacia M.Z., Kochan K., Kaczor A., Baranska M. (2015). Raman spectroscopy of lipids: A review. J. Raman Spectrosc..

[49] Rohleder D., Kiefer W., Petrich W. (2004). Quantitative analysis of serum and serum ultrafiltrate by means of Raman spectroscopy. Analyst.

[50] Wagner M., Ivleva N.P., Haisch C., Niessner R., Horn H. (2009). Combined use of confocal laser scanning microscopy ( CLSM ) and Raman microscopy (RM): Investigations on EPS—Matrix. Water Res..

[51] Keleştemur S., Avci E., Çulha M. (2018). Raman and surface-enhanced Raman scattering for biofilm characterization. Chemosensors.

[52] Ivleva N.P., Wagner M., Horn H., Niessner R., Haisch C. (2010). Raman microscopy and Surface-Enhanced Raman Scattering (SERS) for in situ analysis of biofilms. J. Biophotonics..

[53] Guo J., Cai W., Du B., Qian M., Sun Z. (2009). Raman spectroscopic investigation on the interaction of malignant hepatocytes with doxorubicin. Biophys. Chem..

[54] Verma S.P., Wallach D.F.H. (1977). Raman spectra of some saturated, unsaturated and deuterated C18 fatty acids in the hch-deformation and ch-stretching regions. Biochim. Biophys. Acta.

[55] Pearman W.F., Lawrence-Snyder M., Angel S.M., Decho A.W. (2007). Surface-enhanced raman spectroscopy for in situ measurements of signaling molecules (autoinducers) relevant to bacteria quorum sensing. Appl. Spectrosc..

[56] Schulz H., Baranska M. (2007). Identification and quantification of valuable plant substances by IR and Raman spectroscopy. Vib. Spectrosc..

[57] Szymańska-Chargot M., Chylińska M., Pieczywek P.M., Rösch P., Schmitt M., Popp J., Zdunek A. (2016). Raman imaging of changes in the polysaccharides distribution in the cell wall during apple fruit development and senescence. Planta.

[58] Agarwal U.P. (2006). Raman imaging to investigate ultrastructure and composition of plant cell walls: Distribution of lignin and cellulose in black spruce wood (Picea mariana). Planta.

[59] Synytsya A. (2003). Spectroscopic estimation of feruloyl groups in sugar beet pulp and pectin. Int. Sugar J..

[60] Ramirez-Mora T., Dávila-Pérez C., Torres-Méndez F., Valle-Bourrouet G. (2019). Raman spectroscopic characterization of endodontic biofilm matrices. J. Spectrosc..

[61] Shao J., Lin M., Li Y., Li X., Liu J., Liang J., Yao H. (2012). In vivo blood glucose quantification using raman spectroscopy. PLoS ONE.

[62] Rygula A., Majzner K., Marzec K.M., Kaczor A., Pilarczyk M., Baranska M. (2013). Raman spectroscopy of proteins: A review. J. Raman Spectrosc..

[63] Srivastava A.K., Iconomidou V.A., Chryssikos G.D., Gionis V., Kumar K., Hamodrakas S.J. (2011). International Journal of Biological Macromolecules Secondary structure of chorion proteins of the Lepidoptera Pericallia ricini and Ariadne merione by ATR FT-IR and micro-Raman spectroscopy. Int. J. Biol. Macromol..

[64] Gelder J., Gussem K., Vandenabeele P., Moens L. (2007). Reference database of Raman spectra of biological molecules. J. Raman Spectrosc..

[65] Khatoon Z., Mctiernan C.D., Suuronen E.J., Mah T. (2018). Bacterial bio fi lm formation on implantable devices and approaches to its treatment and prevention. Heliyon.

[66] Jabbouri S., Sadovskaya I. (2010). Characteristics of the biofilm matrix and its role as a possible target for the detection and eradication of Staphylococcus epidermidis associated with medical implant infections. FEMS Immunol. Med. Microbiol..

[67] Wroblewska M., Struzycka I., Mierzwinska-Nastalska E. (2015). Significance of biofilms in dentistry. Przegl. Epidemiol..

[68] Nadell C.D., Xavier J.B., Levin S.A., Foster K.R. (2008). The evolution of quorum sensing in bacterial biofilms. PLoS Biol..

[69] Mitchell K.F., Zarnowski R., Sanchez H., Edward J.A., Reinicke E.L., Nett J.E., Andes D.R. (2015). Community participation in biofilm matrix assembly and function. Proc. Natl. Acad. Sci. USA.

[70] Burmølle M., Webb J.S., Rao D., Hansen L.H., Sørensen S.J., Kjelleberg S. (2006). Enhanced biofilm formation and increased resistance to antimicrobial agents and bacterial invasion are caused by synergistic interactions in multispecies biofilms. Appl. Environ. Microbiol..

[71] Ren D., Madsen J.S., Sørensen S.J., Burmølle M. (2015). High prevalence of biofilm synergy among bacterial soil isolates in cocultures indicates bacterial interspecific cooperation. ISME J..

[72] Rice S.A., Wuertz S., Kjelleberg S. (2016). Next-generation studies of microbial biofilm communities. Microb. Biotechnol..

[73] Flemming H.C., Wingender J. (2010). The biofilm matrix. Nat. Rev. Microbiol..

[74] Flemming H.C., Neu T.R., Wozniak D.J. (2007). The EPS matrix: The “House of Biofilm Cells”. J. Bacteriol..

[75] Matsuyama T., Nakagawa Y. (1996). Surface-active exolipids: Analysis of absolute chemical structures and biological functions. J. Microbiol. Methods.

[76] Ward O.P. (2010). Microbial Biosurfactants and Biodegradation. Adv. Exp. Med. Biol..

[77] Baughn A.D., Rhee K.Y. (2014). Biofilm matix proteins. Microbiol. Spectr..

[78] Arciola R.C., Campoccia D., Speziale P., Montanaro L., William J. (2012). Biomaterials Bio fi lm formation in Staphylococcus implant infections. A review of molecular mechanisms and implications for bio fi lm-resistant materials. Biomaterials.

[79] Nishikawara F., Nomura Y., Imai S., Senda A., Hanada N. (2007). Evaluation of cariogenic bacteria. Eur. J. Dent..

[80] Diem M., Romeo M., Boydston-White S., Miljkovic M., Matthaus M. (2004). A decade of vibrational micro-spectroscopy of human cells and tissue (1994–2004). Analyst.

[81] Zwielly A., Gopas J., Brkic G., Mordechai S. (2009). Discrimination between drug-resistant and non-resistant human melanoma cell lines by FTIR spectroscopy. Analyst.

[82] Guo W., Piao S., Yang T.C., Guo J., Iqbal K. (2020). High-resolution power spectral estimation method using deconvolution. IEEE J. Ocean. Eng..

[83] Morhac M., Matousek V. (2011). High-resolution boosted deconvolution of spectroscopic data. J. Comput. Appl. Math..

[84] Váczi T. (2014). A new, simple approximation for the deconvolution of instrumental broadening in spectroscopic band profiles. Appl. Spectrosc..

[85] Wilson C., Lukowicz R., Merchant S., Valquier-Flynn H., Caballero J., Sandoval J., Clement B. (2017). Quantitative and qualitative assessment methods for biofilm growth: A mini-review. Res. Rev. J. Eng. Technol..

